# P-575. Sexual Behavior and Sexually Transmitted Infection among Sexually Active, Heterosexual Young Adults in Japan: a Cross-Sectional Internet Survey

**DOI:** 10.1093/ofid/ofaf695.790

**Published:** 2026-01-11

**Authors:** Masami Kitaoka, Mizue Kanai, Shingo Nishiki, Ayu Kasamatsu, Takuri Takahashi, Yuzo Arima, Takuya Yamagishi, Tomimasa Sunagawa

**Affiliations:** Field Epidemiology Training Program Japan (FETP-J), National Institute of Infectious Diseases, Japan Institute for Health Security, Shinjuku, Tokyo, Japan; Mie University Graduate School of Medicine, Tsu, Mie, Japan; Japan International Cooperation Agency (JICA), Chiyoda, Tokyo, Japan; Japan Institute for Health Security, Shinjuku, Tokyo, Japan; National Institute of Infectious Diseases, Shinjuku, Tokyo, Japan; Japan Institute for Health Security, Shinjuku, Tokyo, Japan; Japan Institute for Health Security, Shinjuku, Tokyo, Japan; National Institute of Infectious Diseases, Shinjuku, Tokyo, Japan

## Abstract

**Background:**

Epidemiological data on sexual behaviors (SBs) and sexually transmitted infections (STIs) in Japan are scarce. This study aimed to understand SBs and potential risk factors for acquiring STIs among sexually active young adults in Japan.

**Methods:**

A cross-sectional, online survey conducted in December 2024 asked questions about demographic characteristics, SBs, history of STIs, and STI testing. Inclusion criteria were 18–34 years of age, living in Japan, and having a history of heterosexual intercourse and STI testing. The target sample size was 5,000 adults. The proportion of those acquiring an STI in the past 6 months for the following were examined, stratified by sex: the number of sex partners (SPs) and relationship status with SP(s); and, at any time in the past, engaging in commercial sex (CS) and transactional sex.

**Results:**

Among 4,860 respondents (including 923 men and 3,930 women), 750 men and 2,805 women had sexual intercourse in the past 6 months and among those 46% of men and 19% of women had multiple SPs. Among respondents of the men, 2% had provided CS and 29% had received CS, whereas the respective proportions for women were 2% and 1%. Transactional sex with SPs met through SNS, matching apps, or casual pickups occurred in 9% of men and 3% of women. A history of STI was present in 43% of the men and 31% of the women, with chlamydia, gonorrhea, and syphilis being the most common in that order. The proportion of those acquiring an STI in the past 6 months was as follows. Among those whose SP was only a regular partner (men (n=360), women (n=2173)), 6% of men and 2% of women acquired an STI. Among those whose SPs were not limited to a regular partner, 18% of men and 15% of women acquired an STI. Among men who provided CS (n=9), 22% acquired an STI, whereas 16% of men who received CS (n=141) acquired an STI. For women (provided CS (n=32), received CS (n=15)), the respective proportions were 41% and 53%. Among those who reported meeting SPs through SNS, matching apps, or casual pickups and having transactional sex (men(n=48), women (n=49)), 48% of men and 43% of women acquired an STI.

**Conclusion:**

About 10% of sexually active young men in this study reported having transactional sex through SNS or matching apps. Having multiple SPs, CS work, and transactional sex were suggested to be risk factors influencing STI acquisition.

**Disclosures:**

All Authors: No reported disclosures

